# An experimental conflict of interest between parasites reveals the mechanism of host manipulation

**DOI:** 10.1093/beheco/arv200

**Published:** 2015-11-23

**Authors:** Nina Hafer, Manfred Milinski

**Affiliations:** Department of Evolutionary Ecology, Max-Planck-Institute for Evolutionary Biology, August-Thienemann-Straße 2, D-24306 Plön, Germany

**Keywords:** host manipulation, host–parasite interactions, *Schistocephalus solidus*, sequential infection, side effects, three-spined stickleback.

## Abstract

Parasites can increase their host’s predation susceptibility. It is a long-standing puzzle, whether this is caused by host manipulation, an evolved strategy of the parasite, or by side effects due to, for example, the parasite consuming energy from its host thereby changing the host’s trade-off between avoiding predation and foraging toward foraging. Here, we use sequential infection of three-spined sticklebacks with the cestode *Schistocephalus solidus* so that parasites have a conflict of interest over the direction of host manipulation. With true manipulation, the not yet infective parasite should reduce rather than enhance risk taking because predation would be fatal for its fitness; if host behavior is changed by a side effect, the 2 parasites would add their increase of predation risk because both drain energy. Our results support the latter hypothesis. In an additional experiment, we tested both infected and uninfected fish either starved or satiated. True host manipulation should act independently of the fish’s hunger status and continue when energy drain is balanced through satiation. Starvation and satiation affect the risk averseness of infected sticklebacks similarly to that of uninfected starved and satiated ones. Increased energy drain rather than active host manipulation dominates behavioral changes of *S. solidus*-infected sticklebacks.

## Lay Summary

Causing energy drain is enough to fulfill a parasite’s need to change host behavior. A parasite can manipulate host behavior to its own interest either directly or indirectly through increased energy drain driving the host to be risk prone. We can distinguish experimentally between these mechanisms using a potential conflict of interest between 2 simultaneous parasites. We find support for the latter mechanism. An additional experiment with hungry and satiated hosts confirms our interpretation.

## INTRODUCTION

Parasites have the potential to change the behavior of their hosts. They can actively manipulate host behavior thereby improving their own fitness. In complex life cycle parasites, such host manipulation often takes the shape of increased predation susceptibility (Holmes and Bethel 1972; Poulin and Thomas 1999; Moore 2002; Poulin 2010; Moore 2013). However, a similar shift in host behavior can result from side effects of an infection. Most animals are faced with a trade-off between predation avoidance and energy consumption. A parasite, by definition, consumes energy from its host. This can result in significant energetic costs to the host (e.g., Barber and Wright 2008; Lettini and Sukhdeo 2010; Vanacker et al. 2012). Therefore, an infected host needs more energy than an uninfected one, shifting the trade-off away from predation avoidance in favor of feeding. Hence, an infected host could become more prone to predation without any host manipulation that would have evolved specifically to enhance transmission, which could be exploited by parasites (Milinski 1990; Lefèvre et al. 2008). If such a side effect or compensatory response achieves an optimal behavioral change from the parasite point of view, there would be no selection for an additional manipulation mechanism.

Under natural conditions, studying potential host manipulation in hosts infected by a certain parasite is complicated by the fact that hosts rarely harbor only a single parasite. Rather, hosts are normally infected by a multitude of different parasites from the same and/or different species (e.g., Petney and Andrews 1998; Kalbe et al. 2002). These parasites might have the same or different optima when it comes to how their host should behave. If these optima differ, a conflict over host manipulation can ensue and affect host behavior (Rigaud and Haine 2005; Thomas et al. 2010; Thomas et al. 2011). Such a conflict can also occur between parasites of the same species if one has just entered the host and the other is ready for transmission to the next host and accordingly one needs to suppress and the other to enhance predation risk of their shared intermediate host. This conflict has been studied using hosts that were either naturally (Sparkes et al. 2004; Dianne et al. 2010) or experimentally (Dianne et al. 2010; Hafer and Milinski 2015) infected by different stages of the same manipulating parasite species. All these studies found that it was the already infective parasite that dominated the resulting behavior.

*Schistocephalus solidus* has a complex life cycle with 2 intermediate hosts: cyclopoid copepods and three-spined sticklebacks (*Gasterosteus aculeatus*). It reproduces in the gut of birds to which *S. solidus* is transmitted when its stickleback host is eaten by a bird. *Schistocephalus solidus* is well known to be associated with changes in various aspects of host behavior in its stickleback host (Milinski 1990; Barber et al. 1995; Barber and Huntingford 1995; Barber et al. 2000; Barber and Wright 2008; Barber and Scharsack 2010) including increased risk taking in the face of both, the correct subsequent bird host (Giles 1983; Giles 1987a; Barber et al. 2004) and dead-end fish predators (Milinski 1985). Any altered behavior potentially leading to increased predation susceptibility of the host due to true host manipulation rather than side effects should not be visible before parasites reach infectivity. This is usually assumed to occur once parasites have reached a weight of 50mg (Tierney and Crompton 1992). In naturally infected fish, the level of behavioral changes often correlates positively with parasite load and no sudden switch seems to occur as would be expected for host manipulation that abruptly sets in when the parasite reaches 50mg (Giles 1983; Giles 1987b; Godin and Sproul 1988). In experimentally infected sticklebacks, no change in reaction to a fish predator occurs when their parasite is still very small and not yet infective (Aeschlimann et al. 2000). Barber et al. (2004) repeatedly measured the response of experimentally infected sticklebacks to a simulated bird predator. They found no changes prior to when the parasites assumedly reached 50mg, but significant changes thereafter. In laboratory-infected sticklebacks, an activation of the innate immune system coincides with when the parasite reaches infectivity (Scharsack et al. 2007). Parasites could exploit preexisting links between the immune system and the neuronal system of their host to manipulate their behavior (Thomas et al. 2005; Poulin 2010; Adamo 2012; Lafferty and Shaw 2013), for example, infection is associated with altered levels of monoamine in the brain of naturally infected sticklebacks (Øverli et al. 2001). However, the correlative nature of these findings cannot prove any causal link. Accordingly, whether or not altered behavior in *S. solidus*-infected sticklebacks is caused by a side effect via energy drain or active host manipulation that has evolved specifically for this purpose has been the subject of an ongoing debate (Milinski 1990; Barber et al. 1995; Barber and Huntingford 1995; Barber et al. 2000; Barber and Wright 2008; Barber and Scharsack 2010), though the recent literature tends to favor true host manipulation (Barber et al. 2004; Barber and Wright 2008; Barber and Scharsack 2010).

In this study, we take advantage of a potential conflict over host manipulation between infective and not yet infective parasites to solve the puzzle of whether host manipulation by *S. solidus* is true manipulation or the consequence of a side effect. Such a conflict should be abundant in nature where sticklebacks often become infected by multiple parasites that will coexist until their host dies. Several, if not all of them, can become large enough within a single fish to reproduce once they reach their final bird host (e.g., Arme and Owen 1967; Pennycuick 1971; Heins et al. 2002; Heins et al. 2011). A conflict between infective and not yet infective parasites should be mirrored in altered host behavior only if there is true manipulation: If a not yet infective parasite shares a host with an already infective conspecific, it is expected to sabotage the older parasite’s manipulation because any predation at this point would be fatal for it. Hence, we should see a compromise in the fish’s behavior reflecting the conflicting parasite interests. Even if such sabotage was to fail completely, combined active host manipulation of 2 disagreeing parasites should never increase risk taking of their host beyond what an infective parasite would achieve when alone. Any such increase in risk taking is thus likely to be a side effect of enhanced energy drain caused by 2 parasites compared with 1 parasite rather than active host manipulation. In a second experiment, using only singly infected hosts, we investigated the effect of both infective and not yet infective parasites on predation avoidance when fish were either hungry or fed to satiation. If only energy drain is responsible for the alteration in host behavior, satiated fish, irrespective of being parasitized, should behave in a more risk adverse manner than hungry ones. By contrast, true host manipulation should act independently of the fish’s hunger status and continue when energy drain is balanced through satiation. It is in the interest of the infective parasite that its host exposes itself to predation also when it is not hungry.

## MATERIALS AND METHODS

### Hosts

Sticklebacks were bred from fish caught in the Große Plöner See, northern Germany. For experiment 1, fish were about 3 months old at the beginning of the experiment. We used 113 fish from 4 different families. Two weeks prior to the first parasite exposure, they were distributed to 8 different tanks, with 15 fish each, 2 tanks per family. For experiment 2, we used 188 fish from 6 families, which were about 7 months old. We used older (and hence larger) fish in experiment 2 in order to ensure that parasites could reach maturity without potentially compressing the fish’s gut to such an extent that infected fish would be unable to become satiated (Milinski 1985). For both experiments, fish families were randomized with regard to treatment. On the day before the (first) infection, we placed each fish in a separate 16-L tank visually isolated from any other fish. Throughout the experiment, fish remained in this home tank and were fed with bloodworms (*Chironomus* sp.). In experiment 1, fish were fed daily except on the day before and during the experiment. For experiment 2, fish were randomly assigned to 2 different feeding treatments. Prior to the experiment, they were either fed to satiation (“satiated”) or starved for 3 days (“starved”). All experiments were conducted with permission of the “Ministry of Energy, Agriculture, the Environment and Rural Areas” of the state of Schleswig-Holstein, Germany (reference number: V 313-72241.123-34).

### Parasites

*Schistocephalus solidus* were bred in an in vitro system in the laboratory (Smyth 1946; Wedekind 1997) from parents dissected from naturally infected fish caught at the “Neustädter Binnenwasser,” northern Germany. We used 2 different families for each experiment and stored the eggs in the fridge at 4 °C until use. Prior to infection, they were incubated for 3 weeks at 20 °C in the dark and then exposed to light overnight to induce the coracidia to hatch (Dubinina 1980). One coracidium each was administered to lab-bred copepods (*Macrocyclops albidus*). In the copepods, they were allowed to grow for 17 days. After 1–2 weeks, copepods were checked for infection by placing them on a microscope slide. Copepods are translucent allowing identification of an infection visually without having to kill the copepod.

### Treatments

#### Experiment 1

For experiment 1, we conducted 2 rounds of infections, 31 days apart. Infections took place inside the fish’s home tank. When fish were placed in individual home tanks on the day prior to the first infection, the home tank was only half-filled and water was only turned on 2 days after infections had taken place. For the second infection, water was turned off and water levels were lowered on the day before the infection. This prevented copepods from escaping through the outflow before the sticklebacks could have consumed them.

For experimental infections, a stickleback was offered 1 copepod that was either infected (to obtain infected sticklebacks) or not (to obtain sham-infected sticklebacks). Because this was repeated twice, it resulted in 4 different treatments: Fish receiving only uninfected copepods (0_0), fish receiving 1 infected copepod either on day 0 (1_0) or on day 31 (0_1), and an uninfected one on the other day and fish receiving 1 infected copepod on day 0 plus on day 31 (1_1). If a fish received 2 parasites, they always originated from 2 different families. This allowed us to unambiguously determine the infection time for each parasite after dissection in multiply exposed fish by identifying which family they came from using microsatellites (Andris et al. 2012).

In experiment 1, 3 fish died during the experiment (1 uninfected, 2 infected only on day 0) and were hence excluded from the analysis. We pooled fish according to the treatment they resembled according to the types of parasite they contained even if they had received more parasites than had managed to establish themselves. This resulted in a total of 110 fish that we could include in the final analysis (0_0: 36; 1_0: 29; 0_1: 19; 1_1: 26).

#### Experiment 2

In experiment 2, infections took place in the same manner described above for the first round of infections in experiment 1. Fish were either infected or uninfected. In order to test fish with parasites that were either not yet infective or infective, we conducted the behavioral tests at 2 different time points, about 6-week (early) and about 10-week (late) postinfection, respectively. For each test, we used a different set of fish to enable us to measure parasite weight just after the behavioral tests, which requires dissection. In total, we exposed or sham exposed 188 fish (64 sham exposed, 124 exposed, half of each early and late), 4 of which died (1 early, exposed but uninfected; 3 late, 2 exposed but uninfected, 1 sham exposed). One additional fish (late, exposed but uninfected) developed an ectoparasitic fungal infection and was hence excluded. Again, we pooled sham exposed and exposed but uninfected fish. Infection treatment and early and late fish were combined with the feeding treatment (satiated or starved) described above in a fully factorial manner (early: 36 uninfected, satiated, 36 uninfected, starved, 10 infected, satiated, 11 infected, starved; late: 37 uninfected, satiated, 35 uninfected, starved, 8 infected, satiated, 11 infected, starved).

### Behavioral experiments

#### Experiment 1

**Figure 1:**
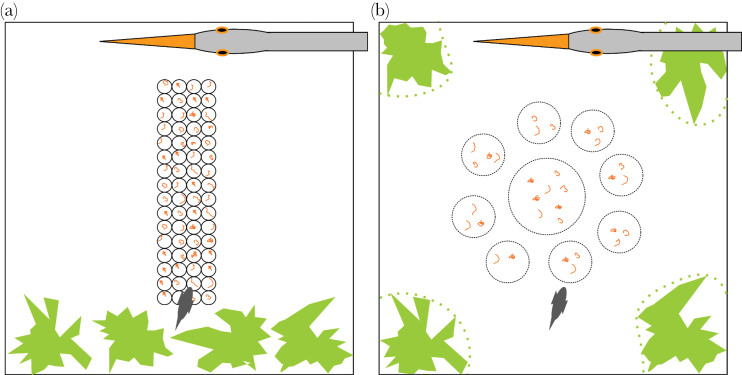
Setup of the experimental tank. (a) Experiment 1 and (b) experiment 2. On one side of the tank, a model heron head was installed in a manner that it could be dropped into the water in a standardized manner to simulate an attack by a heron. In experiment 1 (a), plants for hiding were placed on the opposite end of the tank; for experiment 2 (b), plants were placed in all 4 corners of the tank. A fish was considered hiding if it was within the dashed lines encircling the plants on the video screen. Small feeding pots were placed in the tank in experiment 1. In experiment 2, food was provided in half-buried Petri dishes in the center of the tank. This food was accessible during training but covered with translucent lids during the experiment.

Experiments took place in a separate experimental tank, 44 by 44cm and filled with water to a height of about 20cm. The ground was covered with sand. An array of 4×16 small pots that contained 1 bloodworm each was placed in the middle of the tank embedded in sand (Milinski 1985). On one side of the tank, a model heron head was installed and clamped with a rubber band in a manner that when the rubber band was released, the heron quickly dipped into the water before returning to an upright position to simulate a predation attack (Giles 1983; Giles 1987a; Barber et al. 2004). Opposite to the model heron, 4 plastic water plants were placed in the tank to provide hides (Figure 1a). On one side of the tank, a mirror was placed roughly in a 45° angle that allows recording a side view of the tank while recording from above. A black curtain to minimize disturbance surrounded the entire setup. Above the experimental tank, a HD-camera (MHD-13MG6SH-D, Mintron, Taiwan) was located that allowed us to videotape all behavioral trials and to monitor them on a screen without disturbing the fish. During a few trials problems occurred with the recording. Before we conducted any experiments, we accustomed the fish to the experimental setup and procedure. Prior to the first infection, fish were transferred twice to the experimental tank in groups of 7 or 8 fish and allowed to feed for at least 1h. That way fish knew where to find the food in the experimental tank prior to the actual experiments. Following the first infection and isolation in individual tanks, each fish was accustomed once more alone for 45min to the experimental tank.

The actual behavioral trials consisted of gently transferring a single fish to the experimental tank within a glass pipe filled with water to minimize disturbance. A timer was started as soon as a fish left the glass pipe. Each fish was then allowed to consume 2 food items. As soon as it had done so, a mechanism was released dipping the model heron head into the water. If a fish failed to consume 2 food items, the simulated heron attack took place after 15min. Trials in which fish were hidden underneath the plants at this time were discarded from analysis because this might have prevented them from perceiving the simulated heron attack (Time point 1: 3 fish; Time point 2–5: 5 fish during each time point). After the simulated heron attack, each fish remained in the experimental tank for 5min. Thereafter, it was removed once it had consumed at least 10 food items. Fish that did not consume 10 food items within 15min were removed from the experimental tank. We confirmed (Supplementary Information I, Figure S1) and analyzed how (Supplementary Information III, Figure S4) fish reacted to the simulated predator attack. In nature, a predator that fails to catch a fish it attacked might remain close by for some time, ready to strike again. Hence, following the simulated predator attack, fish should perceive an enhanced predation risk. We recorded when fish resumed feeding after the simulated heron attack, how much food they consumed within the subsequent 5min, and where they consumed the first 2 food items before and after the simulated heron attack. Thereafter, fish were gently returned to their home tank. From the recordings, we estimated the position of each fish every 2s over the course of 5min starting 10s after the simulated heron attack using the manual tracking plugin within ImageJ (Rasband 1997–2015). We conducted trials at 4 different time points. Each of these trials stretches over 5 days. We timed them in a manner that each fish was tested once every 10 days (i.e., once per time point). The first trial took place 7.5 weeks after the first infection (parasite age during trials: parasite from day 0/parasite from day 31: Time point 1: 52–56/21–25 days; Time point 2: 62–66/31–35 days; Time point 3: 72–76/41–45 days; Time point 4: 82–86/51–55 days).

#### Experiment 2

We used the same experimental tank with the same model heron as in experiment 1, but the exact layout of the tank differed and the mirror was absent. Because we had observed no preference for fish to stay away from the heron side of the tank in experiment 1, we equipped the tank more symmetrically in experiment 2. Bloodworms were provided in Petri dishes placed in the center of the tank. During the actual experiments, these Petri dishes were covered with translucent lids. Fish were able to perceive but not to access food to prevent them from becoming satiated. Unlike in experiment 1 where food had been accessible that allowed us to also observe fish behavior before the simulated heron attack without any food sources becoming depleted or fish satiated, but it did not allow us to count the number of food items fish consumed. Plants were provided in all 4 corners (Figure 1b). Four times over the course of about 10 days, fish were transferred to the test tank to become accustomed to this setup. Each fish was allowed to feed for 15–30min. Food inside the Petri dishes was accessible during this training and no additional food was provided in the home tank.

As in experiment 1, fish were transferred to the experimental tank in a glass pipe and a timer started as soon as they left it. We then measured the time fish spent hiding for 5min starting 30s after a fish had left the glass pipe or as soon as a fish emerged from hiding if it had been hiding at that time. A fish was considered hiding as soon as it was partially within immediate proximity to the plants defined by a line had been drawn on the screen prior to the experiments encircling the entire plant but smoothing out its uneven structure (Figure 1b). Once fish had been recorded for 5min, the simulated heron attack occurred. If fish were hiding at that time, we waited until they left the hide. If fish were still hiding 30min after the initial recording started, we discarded them (6 early fish: 2 infected and satiated, 1 infected and starved, 3 uninfected and satiated and 6 late fish: 2 infected and satiated, 4 uninfected and satiated). Starting 10s after the simulated heron attack, we again recorded how much time fish spent hiding and when they first reemerged from hiding. If fish did not reemerge from hiding within 15min, we stopped the trial.

### Dissection

After the experiment (i.e., after the fourth time point [experiment 1] or directly after the behavioral trial [experiment 2]), fish were killed by placing them in an overdose of an anesthetic MS222. Their body cavity was opened and any parasite found was removed from the body cavity and weighted.

To determine when parasites might have reached infectivity in experiment 1, we used their known age and weight at dissection to estimate when they would reach 50mg, which is when they are assumed to become infective to birds (Supplementary Information II, Figure S2).

### Statistical analysis

All statistical analysis took place and all plots were created in R (R Development Core Team 2010). We only present relevant *P* values in the Results section for better readability. For exact statistical outputs, please refer to Tables 1–5.

**Table 1:** Outcome of likelihood ratio tests

Factors	Total amount of food consumed within 5 min			Fish activity (average distance moved within 2s)			Average position where the first 2 food items before and after the simulated heron attack were consumed		
	df	χ^2^	*P*	df	χ^2^	*P*	df	χ^2^	*P*
+Treatment	8,3	17.968	0.0005	9,3	2.279	0.5165	23,3	1.891	0.5953
+Treatment:time point	11,3	15.221	0.0016	12,3	2.763	0.4296	32,9	12.196	0.2025
+Treatment:time interval							35,3	1.7218	0.6321
+Treatment:time point: time interval							47,12	13.258	0.3505
	422 observations on 110 fish			405 observations on 110 fish			518 observations 89 on fish		

df, degrees of freedom. For the number of food items consumed and the feeding position, we used generalized linear mixed models with Poisson error family. For fish activity, we used linear mixed models after log-transforming the data. We included time point as fixed factor and used fish identity as random effects including the repeat to account for the presence of intraindividual variation between repeats. For the feeding position, we additionally included the time interval in the recording (i.e., before vs. after the simulated heron attack) both in the random effects and as a fixed effect. Subsequently, we added the treatment and its interactions with repeat. Test statistics and Markov chain Monte Carlo-estimated *P* values are for the comparison with the preceding model.

**Table 2:** Outcome of multiple comparisons for each time point for the latency to resume feeding

Comparison		Time point 1			Time point 2			Time point 3			Time point 4		
Treatment 1	Treatment 2	χ^2^	*P*	*P* adj^a^	χ^2^	*P*	*P* adj^a^	χ^2^	*P*	*P* adj^a^	χ^2^	*P*	*P* adj^a^
0_0	1_0	0.48	0.9242	1	22.50	**0.0001**	**0.0003**	14.17	**0.0027**	**0.0161**	11.30	**0.0102**	0.0612
0_0	0_1	0.01	0.9997	1	12.26	**0.0065**	**0.0392**	9.68	**0.0215**	0.1292	4.63	0.2009	1.2051
0_0	1_1	13.11	**0.0044**	**0.0265**	30.80	**<0.0001**	**<0.0001**	42.95	**<0.0001**	**<0.0001**	54.11	**<0.0001**	**<0.0001**
0_1	1_0	0.45	0.9292	1	0.34	0.9515	1	0.01	0.9997	5.9984	0.73	0.8652	5.1911
1_0	1_1	8.60	**0.0351**	0.2103	1.68	0.6410	1	10.01	**0.0185**	0.1108	19.45	**0.0002**	**0.0013**
0_1	1_1	10.54	**0.0145**	0.0868	2.97	0.3961	1	8.73	**0.0330**	0.1983	23.97	**<0.0001**	**0.0002**

We conducted survival analysis including only 2 treatments at once. Significant *P* values are highlighted in bold. 0_0: Fish not infected by *Schistocephalus solidus*; 1_0: Fish singly infected on day 0; 0_1: Fish singly infected on day 31; 1_1: Fish sequentially infected on day 0 plus on day 31. If there were differences between treatments, treatment 1 is the one with the longer time to resume feeding within each comparison, that is, the more risk-averse fish.

^a^Adjusted *P* values represent Bonferroni correction for multiple testing.

**Table 3:** Post hoc comparison between treatments for the amount of food consumed within 5min after the simulated heron attack

Comparison		Time point 1		Time point 2		Time point 3		Time point 4	
		*t*	*P*	*t*	*P*	*t*	*P*	*t*	*P*
0_0	1_0	0.940	0.7808	−4.054	**0.0002**	−7.598	**<0.0001**	−6.031	**<0.0001**
0_0	0_1	0.871	0.8176	−2.095	0.1526	−5.691	**<0.0001**	−3.482	**0.0030**
0_0	1_1	−3.831	**0.0006**	−7.298	**<0.0001**	−9.111	**<0.0001**	−7.985	**<0.0001**
0_1	1_0	0.050	1	1.421	0.4829	1.297	0.5606	2.049	0.1674
1_0	1_1	−4.425	**0.0001**	−3.419	**0.0032**	−1.778	0.2804	−2.292	0.0980
0_1	1_1	−3.869	**0.0006**	−4.237	**0.0002**	−2.809	**0.0250**	−3.946	**0.0005**

We used Tukey’s test using general linear hypotheses. Fish identity and repeat were included as random effect. Significant *P* values are highlighted in bold. 0_0: Fish not infected by *Schistocephalus solidus*; 1_0: Fish singly infected on day 0; 0_1: Fish singly infected on day 31; 1_1: Fish sequentially infected on day 0 plus on day 31. If there were differences between treatments, treatment 1 is the one with the lower number of food items consumed within each comparison, that is, the more risk-averse fish.

**Table 4:** Analysis of the time spent hiding before and after a simulated heron attack

Factors	Early (6-week postinfection)			Late (6-week postinfection)		
	df	χ^2^	*P*	df	χ^2^	*P*
+Time interval	4,1	81.225	<0.0001	4,1	48.537	<0.0001
+Infection	5,1	0.002	0.9661	5,1	0.001	0.9811
+Time interval:infection	6,1	0.015	0.9026	6,1	0.529	0.4671
+Feeding	7,1	19.751	<0.0001	7,1	29.357	<0.0001
+Feeding:time interval	8,1	38.144	<0.0001	8,1	28.876	<0.0001
+Feeding:infection	9,1	0.047	0.8292	9,1	0.116	0.7337
+Feeding:infection:time interval	10,1	0.156	0.6932	10,1	0.393	0.5309
	174 observations on 87 fish			168 observations on 84 fish		

df, degrees of freedom. The table presents the outcome of likelihood ratio tests. We used linear mixed models after log-transforming the data. We included fish identity as random effects. Subsequently, we added the time interval in the recording (before vs. after the simulated heron attack), the feeding treatment, the infection treatment, and all their interactions. Test statistics and Markov chain Monte Carlo-estimated *P* values are for the comparison with the preceding model.

**Table 5:** Post hoc comparison between treatments and time interval (before vs. after heron) for the time fish spend hiding

Comparison	Early (6-week postinfection)		Late (10-week postinfection)	
	*t*	*P*	*t*	*P*
Before the simulated heron attack: starved—satiated	−7.698	**<0.0001**	−8.142	**<0.0001**
After the simulated heron attack: starved—satiated	0.031	1	−1.206	0.6184
Hungry: before the simulated heron attack—after the simulated heron attack	−14.778	**<0.0001**	−10.973	**<0.0001**
Full: before the simulated heron attack—after the simulated heron attack	−4.562	**<0.0001**	−2.212	0.1176
Before the simulated heron attack, starved—after the simulated heron attack, satiated	−11.453	**<0.0001**	−10.059	**<0.0001**
Before the simulated heron attack, satiated—after the simulated heron attack, starved	−3.785	**0.0009**	−0.710	0.8913

We used Tukey’s test using general linear hypotheses. Fish identity was included as random effect. Significant *P* values are highlighted in bold.

#### Experiment 1

To investigate the latency with which fish resumed feeding after the simulated heron attack, we performed a survival analysis by fitting a parametric survival regression model for each time point. We used the survreg function in the survival package (Therneau 2014) with Weibull distribution and the time to emerge from hiding as response. For fish that did not emerge within 15min, we set this time to 15min (900s). We additionally included whether an event occurred (fish emerged from hiding) or not (data censored after 15min) into the response. To investigate the effect of each treatment more closely, we conducted pairwise comparisons, using the same models but including data from only 2 treatments at each time with Bonferroni corrections.

We used generalized linear mixed models in the lme4 package (Bates et al. 2014) with Poisson error family to analyze how much food fish consumed and where they fed on average. To analyze fish activity (i.e., the average distance fish moved within 2s), we used linear mixed models (lme4 package) (Bates et al. 2014) after log-transforming the data. We included fish identity as a random factor to account for variation between fish. Time point was included within the fish identity random effect to account for variation within fish between days. Time point was also added as fixed factor to account for the change over time we expected to occur. For the position where fish fed, we included the time interval in the recording (i.e., before vs. after the simulated heron attack) both in the random factor and as a fixed effect. We then stepwise included treatment and its interaction with time point and, if appropriate, the time interval and its interaction. Subsequently, we performed likelihood ratio tests to compare models. A model was accepted if it was significantly better than a less complex model at explaining the data. For each time point, we performed a separate Tukey’s test using general linear hypotheses within the multcomp package (Hothorn et al. 2008) to determine when treatments differed.

#### Experiment 2

We analyzed fish from the early and late group separately. To investigate the fish’s latency to reemerge from hiding after the simulated heron attack, we again fitted a parametric survival regression model in the survival package using the survreg function (Therneau 2014). Similarly to described above, we used the time to emerge from hiding (set to 15min if fish failed to remerge) and whether or not they did emerge as response.

To analyze how much time fish spent hiding, we log-transformed the data and then applied linear mixed models from the lme4 package (Bates et al. 2014) using fish identity as random factor and the time interval (i.e., before vs. after the simulated heron attack) as fixed effect. For both models, we stepwise added feeding treatment, infection and time interval (only for time hiding), and all 2-way interactions. Subsequently, we performed likelihood ratio tests (see above).

## RESULTS

### Experiment 1

Treatment significantly influenced how long it took fish to resume feeding after the simulated heron attack during all 4 time points (Time point 1: $\chi_{\text{1}0\text{4},\text{3}}^{2}$ = 14.96, *P* = 0.0019; Time point 2: $\chi_{\text{1}03,\text{3}}^{2}$ = 42.82, *P* < 0.0001; Time point 3: $\chi_{\text{1}02,\text{3}}^{2}$ = 46.79, *P* < 0.0001; Time point 4: $\chi_{\text{1}01,\text{3}}^{2}$ = 55.09, *P* < 0.0001). Likewise, treatment (*P* = 0.0005) and its interaction with time point (*P* = 0.0002) influenced how much food fish consumed in the 5min following the simulated heron attack (Table 1). Neither treatment nor its interaction with time point had any effect on how far fish moved or where they fed (*P* > 0.2, Table 1). Accordingly, we focus on the latency to resume feeding and the amount of food fish consumed, and conduct post hoc tests to disentangle when and between which treatments significant differences occurred.

#### Is there predation suppression?

Fish with not yet infective parasites (i.e., before Time point 2 in fish infected on day 0 and before Time point 4 in fish infected on day 31, see Supplementary Information II) never took significantly longer to resume feeding (*P* > 0.9, Table 2) or consumed less food than uninfected fish (*P* > 0.1, Table 2). If there were any differences, fish with not yet infective parasites were even more risk prone than uninfected fish (*P* < 0.04, Tables 2 and 3).

#### Is there predation enhancement?

**Figure 2:**
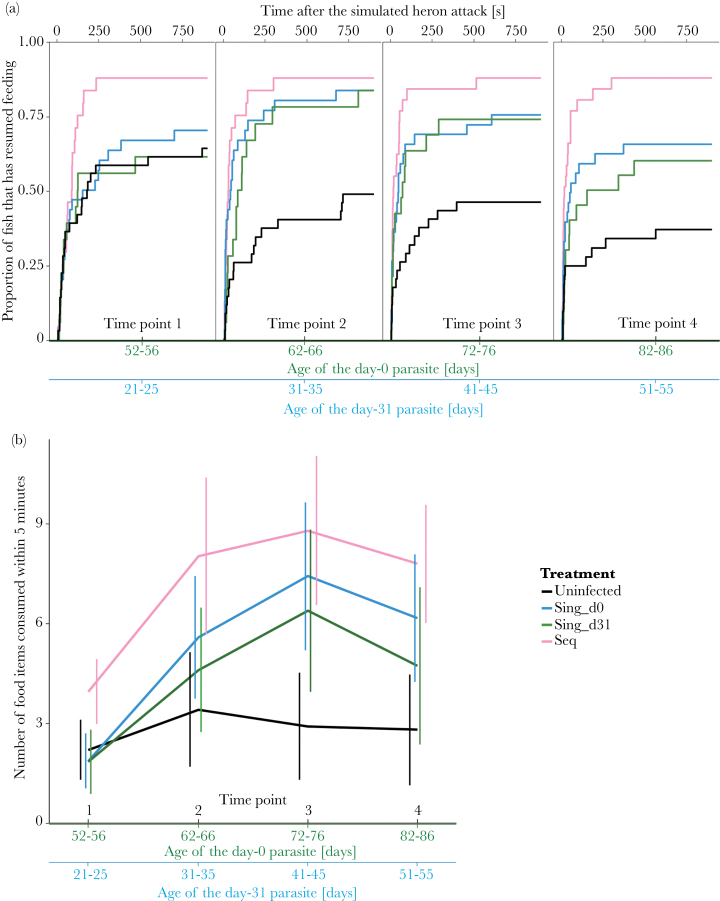
Behavioral observations after a simulated heron attack. (a) Latency to resume feeding. (b) Number of food items consumed within 5min. Bold numbers on the *x* axis indicate that a parasite of that age was infective. 0_0: Fish not infected by any parasite; 0_0: Fish not infected by *Schistocephalus solidus*; 1_0: Fish singly infected on day 0; 0_1: Fish singly infected on day 31; 1_1: Fish sequentially infected on day 0 plus on day 31. *N*: Time point 1: 0_0: 35, 1_0: 29, 0_1: 19, 1_1: 24; Time point 2: 0_0: 34, 1_0: 29, 0_1: 18, 1_1: 24; Time point 3: 0_0: 33, 1_0: 28, 0_1: 18, 1_1: 26; Time point 4: 0_0: 31, 1_0: 29, 0_1: 19, 1_1: 26.

Once *S. solidus* is infective (on average from Time point 2 or 3 onwards for those in fish infected only on day 0 and during Time point 4 for those in fish infected only on day 31, see Supplementary Information II), it should increase the predation susceptibility of its host and thereby enhance transmission. From Time point 2 onwards, fish infected on day 0 resumed feeding sooner than uninfected fish (*P* < 0.02, Table 2, Figure 2a), though this was only a trend during the fourth time point (*P* = 0.0612, Table 2), and consumed more food (*P* < 0.0003, Table 3, Figure 2b). Surprisingly, fish infected on day 31 also started to increase their risk taking during the second time point. They resumed feeding significantly sooner than uninfected fish during the second time point (*P* = 0.0392, Table 2, Figure 2a) and consumed more food from Time point 3 onwards (*P* < 0.004, Table 3, Figure 2b). Hence, we did see increased risk taking likely to result in predation enhancement. It seems to occur about the same time in fish infected on day 31 and day 0 even so parasites in fish infected on day 31 are younger and become infective later. There are 2 different but not mutually exclusive explanations for these findings that we discuss further in the Supplementary Information II.

#### Is there conflict?

We did not find any significant differences for any of the traits we measured between fish singly infected either on day 0 or on day 31 (*P* > 0.4, Tables 2 and 3, Figure 2), which would predict a conflict between parasites when together in a fish.

#### What is the outcome of sequential coinfections?

Sequentially infected fish resumed feeding significantly sooner than uninfected fish (*P* < 0.03, Table 2, Figure 2a) and ate more food (*P* < 0.0007, Table 3, Figure 2b) during each time point. They also resumed feeding significantly sooner than fish infected only on day 31 (*P* = 0.0002, Table 2, Figure 2a) during the fourth time point and consumed significantly more food throughout the experiment (*P* < 0.03, Table 3, Figure 2b). They even resumed feeding sooner during Time point 4 (*P* = 0.0013, Table 2, Figure 2a) and consumed significantly more food during Time points 1 and 2 (*P* < 0.004, Table 3, Figure 2b) than fish infected only on day 0. During this time the parasite from day 31 in sequentially infected fish could not yet have been infective.

### Experiment 2

#### Effect of parasite infection

**Figure 3:**
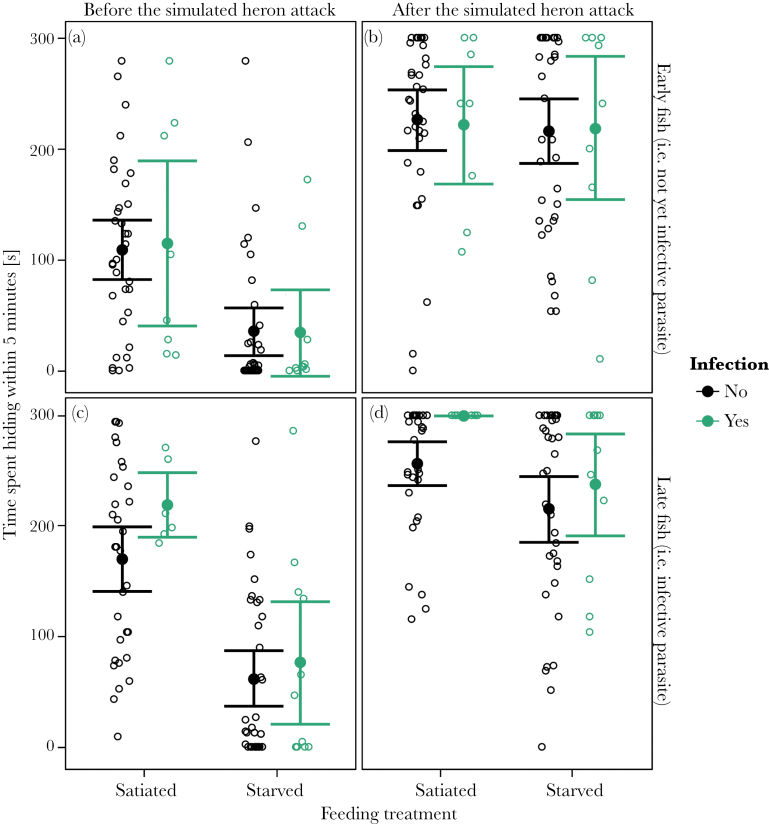
Time spent hiding before (a, c) and after (b, d) a simulated heron attack for early (a, b) and late fish (c, d). We recorded the time fish spent hiding for 5min starting 30s after the fish had entered the tank or, if it was hiding at that time, once it had left hiding (before the simulated heron attack) and for 5min starting 10s after the simulated heron attack irrespective of whether fish were hiding at that time or not (after the simulated heron attack). Error bars indicate 95% confidence interval. *N*: Early: satiated: uninfected: 33, infected: 8, starved: uninfected: 36, infected: 10; Late: satiated: uninfected: 33, infected: 6, starved: uninfected: 35, infected: 11.

In the early group (i.e., prior to reaching infectivity), infection did not significantly affect the fish’s latency to reemerge from hiding ($\chi_{85,\text{1}}^{2}$ = 1.459, *P* = 0.2271). In the late group (i.e., after reaching infectivity), infection did have a significant effect on when fish reemerged from hiding ($\chi_{82,\text{1}}^{2}$ = 10.511, *P* = 0.0012). Contrary to the manipulation hypothesis, infected fish were less likely to reemerge than uninfected fish. The time fish spend hiding both before and after the simulated heron attack was never significantly affected by infection (*P* > 0.9, Table 4, Figure 3).

#### Effect of feeding treatment

How long after the simulated heron attack, fish reemerged from hiding was not affected by the feeding treatment in early fish ($\chi_{84,\text{1}}^{2}$ = 1.238, *P* = 0.2659), but in late fish, starved fish emerged sooner ($\chi_{81,\text{1}}^{2}$ = 13.815, *P* = 0.0002). The time fish spent hiding was affected by the feeding treatment and its interaction with the time interval (before vs. after the simulated heron attack) both in early and late fish (*P* < 0.0001, Supplementary Table S6). Post hoc tests revealed that starved fish spent more time hiding but only before the simulated heron attack (*P* < 0.0001, Table 5, Figure 3a,c) and not thereafter (*P* > 0.6, Supplementary Table S7, Figure 3b,d).

There was no significant interaction between infection and feeding treatment, neither for when fish reemerged from hiding (early: $\chi_{83,\text{1}}^{2}$ = 1.113, *P* = 0.2915, late: $\chi_{80,\text{1}}^{2}$ = 3.422, *P* = 0.064) nor for the amount of time they spend hiding (*P* > 0.7, Table 4). The risk averseness of sticklebacks seems similarly affected by starvation in both infected and uninfected fish.

## DISCUSSION

We test experimentally whether host manipulation by a parasite is due to active manipulation that has evolved for this purpose or caused by a side effect of the parasite draining energy from the host, thus forcing it to change its trade-off between feeding and avoiding predation toward feeding (Milinski 1990; see also Lefèvre et al. 2008). If 2 parasites with different interests share the same host, there is potential for conflict. One parasite might win this conflict (Sparkes et al. 2004; Dianne et al. 2010; Hafer and Milinski 2015), but the other parasite should never enhance the winning parasite’s manipulation. However, in the present study, the losing parasite enhances the winner’s manipulation when three-spined sticklebacks were experimentally infected by 2 *S. solidus* at different times. Fish infected by both an already infective and a not yet infective *S. solidus* show a stronger reduction in risk averseness than fish infected by either parasite alone. Bird predation on their shared host will allow the infective parasite to complete its life cycle and reproduce, but for the not yet infective parasite, it will be fatal. It cannot reproduce, yet. Why should the not yet infective *S. solidus* enhance manipulation that is potentially fatal to it? No active host manipulation should evolve to such an effect. However, active host manipulation is not the only way by which *S. solidus* could affect its host’s behavior; it also drains substantial amounts of energy from it (Walkey and Meakins 1970; Barber and Wright 2008), forcing it to consume more food even if this comes at the cost of exposing itself to increased predation. Such energy drain will also be exerted by a not yet infective *S. solidus*. Energy drain can, unlike true host manipulation, explain why fish infected with both an infective and a not yet infective parasite behave in a more risk prone manner than those infected by the infective *S. solidus* only. Thus, this experiment shows that the not yet infective *S. solidus* does not manipulate the stickleback’s behavior.

Normally 50mg (see Tierney and Crompton 1992) are assumed to present a threshold under which *S. solidus* cannot reproduce; but this might be somewhat arbitrary. Indeed, even *S. solidus* smaller than 50mg can establish themselves in a bird, albeit this is very rare (Tierney and Crompton 1992). In addition, if sharing with an already infective conspecific, the not yet infective *S. solidus* is more likely to be eaten by a bird too early. It would hence benefit from becoming infective at a smaller size. This could then offer an alternative explanation for why double-infected hosts were more risk prone—what we measured might have been cooperation enhancing (true) host manipulation rather than inevitable energy drain. However, at least during our first 3 time points, nearly all parasites from day 31 should have been smaller than even 25mg. Tierney and Crompton (1992) observed no successful establishment of *S. solidus* below that size. Given that they used naturally infected hosts, it seems likely that at least some of their parasites, too, would have originated from sequential infections.

In order to test the hypothesis that energy drain rather than true host manipulation causes behavioral alterations in *S. solidus*-infected sticklebacks further, we compared fish with not yet infective or infective parasites to uninfected fish when they had either been starved for 3 days or fed to satiation. In our first experiment, only fish that had been starved for 2 days have been used to test the effect of *S. solidus* on host behavior. The most decisive experiment to test for true host manipulation versus increased energy drain, however, is to test satiated fish. Increased energy drain should not increase the risk taking of fish that do not require any additional energy because they are satiated. By contrast, host manipulation that has evolved for this specific purpose should be independent of hunger levels and also alter the risk taking of satiated fish. In the second experiment, we do not observe any altered behavior due to a parasite infection in fish that have been fed to satiation. Thus, also infective *S. solidus* do not manipulate their stickleback host’s behavior. Previous studies that have tested for an effect of satiation on host manipulation by *S. solidus* have reported that also naturally infected fish seem to act just as risk averse as uninfected ones when satiated (Giles 1987a; Barber et al. 1995). This is in perfect agreement with our hypothesis that host manipulation in *S. solidus*-infected sticklebacks is due to increased energy drain but inconsistent with active host manipulation.

Surprisingly, unlike in experiment 1 and other previous studies (Giles 1983; Giles 1987a; Barber et al. 1995; Barber and Huntingford 1995; Barber et al. 2004), we do not observe any effect of *S. solidus* infection even in fish that have been starved for 3 days. In heavily infected fish, *S. solidus* probably compresses the gut to such an extent that there is not enough space left for food preventing such fish from ever becoming satiated (Milinski 1985; Cunningham et al. 1994), this is again a side effect caused by the parasite and no active manipulation. To avoid this side effect in experiment 2 that depended on having infected fish that could become fully satiated, we used older (and hence larger) fish. This however could have caused differences between experiments 1 and 2. Older fish react more risk averse to a simulated bird attack (Krause et al. 1998). In juvenile fish, parasites affect host performance more easily because even uninfected juveniles might be closer to their physiological and morphological limits than adults (McElroy and de Buron 2014). Even in uninfected fish, starvation affects small fish more severely than larger fish (Ivlev 1961; Krause et al. 1998). This might render juvenile fish particularly prone to energy drain. This together with larger relative parasite sizes in juvenile fish could have rendered fish in our first experiment more prone to behavioral changes caused by side effects than those in our second experiment (Supplementary Information II). This interpretation does not affect our conclusions from the experiment with satiated fish: Active host manipulation, however, should not stop in larger and older hosts. Previous studies that reported a lack of host manipulation in satiated fish have reported its existence in hungry fish (Giles 1987a; Barber et al. 1995), which is in line with our interpretation, that is, that host manipulation is caused by enhanced energy drain.

Even if apparent host manipulation is caused by side effects, selection might still act on it. Selection will favor behavioral changes that enhance transmission at the right time and select against traits that do not, irrespective of their underlying mechanisms (Milinski 1990; Thomas et al. 2005; Lefèvre et al. 2008; Poulin 2010; Moore 2013). In the present study, several aspects of host manipulation by *S. solidus* in its stickleback host appear suboptimal. Host manipulation should set in once an optimal time for transmission is reached (Hammerschmidt et al. 2009; Parker et al. 2009). *Schistocephalus solidus* is only rarely able to become reproductive before reaching roughly 50mg in its fish host (Tierney and Crompton 1992). Accordingly, any increase in predation susceptibility before that time would not be adaptive in terms of transmission, though it might be adaptive to some extend because the parasite needs the fish to provide extra energy. If *S. solidus* increases hunger levels by restricting the space in the body cavity (Milinski 1985; Cunningham et al. 1994), this might cause in addition to energy drain satiation independent extra apparent host manipulation once *S. solidus* has reached a certain relative size compared with its host. In nature, hosts are usually much smaller than those that we used to avoid the compression effect in our second experiment. Copepods have the optimal prey size for juvenile sticklebacks; large sticklebacks are less likely to attack copepods, the first intermediate host of *S. solidus* (Christen and Milinski 2005). From the infective parasite’s point of view, it would also be ideal if host manipulation is independent of hunger levels because this would increase predation even more. In the laboratory, conditions can often be very benign and food readily available. By contrast, in nature, complete satiation might be much rarer especially in infected fish whose competitive ability is impaired (Milinski 1986; Barber and Ruxton 1998). In addition, as long as hosts have the usual small size, their hunger is maintained at a high level because of the parasite’s compressing their gut. There might not be much potential for improving apparent manipulation by additional true host manipulation.

Our results strongly suggest that apparent host manipulation by *S. solidus* in its stickleback host occurs as inevitable side effect of infection. Through draining energy from the host and restricting space in the gut, the parasite moves the fish’s trade-off between feeding and avoiding predation (Milinski and Heller 1978) toward feeding thus exposing it to predators. Selection might not be able to improve the resulting “manipulation” effect by adding an extra mechanism. The not yet infective parasite appears to be the loser. Multiple infections of *S. solidus* in three-spined sticklebacks are frequent in nature (Arme and Owen 1967; Heins et al. 2002). Selection should favor parasites that can counteract enhanced predation susceptibility before they reach infectivity. This would require a true manipulation mechanism, which does not seem to exist. On the contrary, through its additional energy drain, a not yet infective parasite aggravates its problem. In the first intermediate host, the copepod, the not yet infective *S. solidus* actively suppresses predation risk, but only when alone. A co-infecting infective *S. solidus* sabotages the not yet infective parasite’s manipulation (Hafer and Milinski 2015), which is the loser again. In the present study, we found that the not yet infective *S. solidus* does not reduce predation risk of its stickleback host, not even when it is alone, depicting a new puzzle. On infection, the stickleback is normally too small to allow *S. solidus* to grow large enough to become infective. Therefore, it has to restrict its growth to allow the fish to grow until big enough (Christen and Milinski 2005). Letting the fish follow its optimal growth strategy and risk taking might thus prevent the parasite from manipulative interference. No true manipulation of stickleback behavior seems to be adaptive for both not yet infective and infective *S. solidus*, side effects of infection fulfill the latter’s needs.

## SUPPLEMENTARY MATERIAL

Supplementary material can be found at http://www.beheco.oxfordjournals.org/

## FUNDING

This work was supported by the Max Planck Society. N.H. received her funding through the International Max Planck Research School (IMPRS) for Evolutionary Biology.

## Supplementary Material

Supplementary Data
