# Supplementary material for: An experimental conflict of interest between parasites reveals the mechanism of host manipulation
Source: Behav Ecol. 2015 Nov 23;27(2):617–27. doi: 10.1093/beheco/arv200 (PMC4797381; doi:10.1093/beheco/arv200)
Supplement: Supplementary Data [file supp_27_2_617__index.html]

An experimental conflict of interest between parasites reveals the mechanism of host manipulation — An experimental conflict of interest between parasites reveals the mechanism of host manipulation — Supplementary Data 

# An experimental conflict of interest between parasites reveals the mechanism of host manipulation

## Supplementary Data

Data files

- Supplementary Data - Supplementary Data
