# Supplementary material for: An experimental conflict of interest between parasites reveals the mechanism of host manipulation
Source: Behav Ecol. 2015 Nov 23;27(2):617–27. doi: 10.1093/beheco/arv200 (PMC4797381; doi:10.1093/beheco/arv200)
Supplement: Supplementary Data [file supp_arv200_SI.doc]

## Supplementary information I: Reaction to the simulated heron attack

To confirm that the fish reacted to the simulated heron attack, we compared the time between feeding successive food items for the first 5 food items a fish consumed. We only used fish that had consumed at least 5 food items. The simulated heron attack occurred once two food items had been consumed. We used a paired Wilcox test in R with Bonferroni corrections to account for multiple testing (R Development Core Team 2010). We did this for each of the four time points separately.

The fish’s latency to resume feeding after the simulated heron attack was significantly greater than either the latency to consume the second food item prior to the simulated heron attack or the latency to consume additional food items once feeding had been resumed during all time points (p<0.0001, Figure S1, Table S1). Clearly, fish perceived the simulated heron attack and reacted to it as a frightening event throughout the entire experiment

**Figure S1: Confirmation of the initial reaction to the simulated heron attack: Latency to consume a subsequent food item.** Note that the latency to consume subsequent food items increases after the simulated heron attack (marked by the vertical red line). Error bars present 95% CI. N: Time point 1:56, Time point 2:65, Time point 3:65, Time point 4:62.

**Table S1: Pairwise comparison for the time taken to feed successive food items.** Significant p-values (α<0.05) have been marked in bold. The simulated heron attack occurred once two food items had been consumed.

| Time point | Number of food items already consumed | | N | Test statistics^1^ | | |
| --- | --- | --- | --- | --- | --- | --- |
|  |  |  |  | v | p | p adjusted^2^ |
|  | | | | | | |
| 1 | 1 | 2 | 56 | 0 | **<0.0001** | **<0.0001** |
| 1 | 2 | 3 | 56 | 1537 | **<0.0001** | **<0.0001** |
| 1 | 3 | 4 | 56 | 747 | 0.9725 | 1 |
|  | | | | | | |
| 2 | 1 | 2 | 65 | 0 | **<0.0001** | **<0.0001** |
| 2 | 2 | 3 | 65 | 2100 | **<0.0001** | **<0.0001** |
| 2 | 3 | 4 | 65 | 616 | **0.0180** | 0.0540 |
|  | | | | | | |
| 3 | 1 | 2 | 65 | 0 | **<0.0001** | **<0.0001** |
| 3 | 2 | 3 | 65 | 2076. | **<0.0001** | **<0.0001** |
| 3 | 3 | 4 | 65 | 1007 | 0.3607 | 1 |
|  | | | | | | |
| 4 | 1 | 2 | 62 | 0 | **<0.0001** | **<0.0001** |
| 4 | 2 | 3 | 62 | 1816 | **<0.0001** | **<0.0001** |
| 4 | 3 | 4 | 62 | 750 | 0.2255 | 0.6765 |

1: Values represent V statistic and p values from a paired Wilcox test

2: Bonferroni correction for multiple testing

## Supplementary information II: Parasite growth & parasite index

### Parasite growth during experiment 1

Since we wanted to measure the behavior of each fish repeatedly, measuring directly when parasites would become infective was impossible in our study. This seems to vary between studies (Barber and Svensson 2003; Scharsack et al. 2004; Scharsack et al. 2007). We made use of the fact that *S. solidus* is usually only able to reproduce once it has reached 50 mg (Tierney and Crompton 1992) and of previously estimated growth curves (Barber and Svensson 2003; Scharsack et al. 2004; Scharsack et al. 2007) to calculate at what age parasites in each of our treatment would become infective in our study.

To obtain growth estimate for each parasite throughout the experiment we used the Nonlinear Least Squares function in the stats package in R (R Development Core Team 2010). Following know estimates of the growth of *S. solidus* (Barber and Svensson 2003; Scharsack et al. 2004; Scharsack et al. 2007), we assumed it to roughly follow a logistic growth and hence used the formula for logistic growth: $y=\frac{a}{1+e^{b*(x-c)}}$. We obtained parasite weight and appropriate parasite age from the literature (Barber and Svensson 2003; Scharsack et al. 2004; Scharsack et al. 2007) and used this data to obtain values for *a*, *b* and a starting value for *c*. We then inserted our known parasite weight and age at dissection and a parasite weight of 0 at infection into the formula to obtain an individual value for c for each parasite. The formulas we thereby obtained could be used to estimate the weight of each parasite during each time point since we knew parasite age. Since we found no appropriate data on growth in *S. solidus* sharing their host with a conspecific, we had to assume that it resembled that of parasites not sharing their host, even so this probably represents a simplification.

To compare parasite weight between different treatments, we used parasite weight at dissection. We fitted an analysis of variance model (ANOVA) in R (R Development Core Team 2010) using parasite weight as response and a factor that combined treatment and parasite age as fixed effect. Since the ANOVA showed a significant effect of our treatment/ parasite age factor, we subsequently conducted a Tukey post hoc test.

Treatment and parasite age combined had a significant effect on parasite weight (F_96,3_=60.56, p<0.0001). A post hoc test revealed that whether sharing their host with a conspecific or not, parasites that infected their host on day 31 were always smaller than those from day 0 (Figure S2, p<0.0001). Parasites that infected their host on day 31 grew significantly smaller when they had to share their host with an older conspecific than when they were alone (Figure S2, p<0.0001). By contrast, parasites that infected their host on day 0, did not significantly differ in their weight whether they had to share their host or not (Figure S2, p=0.8167).

**Figure S2: Estimated parasite growth curves.** The shape of the growth curves was obtained from the literature (Barber and Svensson 2003; Scharsack et al. 2004; Scharsack et al. 2007) and in combination with parasite weight and age at dissection used to estimate an average growth curve for each treatment. Actual parasite weight was only measured at dissection (dark grey bar). Error bars indicate 95% CI. The X-axis indicates the age of the parasite from day 0. Parasites that infected their host on day 31 are always 31 days younger. The horizontal black line indicates the 50 mg threshold below which reproduction inside the definite bird host is rare (Tierney and Crompton 1992). The light grey bars indicate the time during which the behavioral measurements for the corresponding time point took place. 1_0; Parasite from day 0: Parasites that infected their host on day 0, 0_1; Parasite from day 31: Parasites that infected their host on day 31, 1_1; Parasite from day 0: Parasites that infected their host on day 0 and had to share with another parasite, 1_1; Parasite from day 31: Parasites that infected their host on day 31 and had to share with another parasite. Parasites from day 0 and day 31 from 1_1 steamed from the same sequentially infected fish. N: 1_0: 29, 0_1:19, 1_1:26.

Reproduction in the definitive bird host is usually assumed to be possibly only after *S. solidus* has reached a weight of at least 50 mg in the fish (Tierney and Crompton 1992). Adaptive host manipulation enhancing the fish’s predation susceptibility should only occur thereafter. Our estimated growth curved allow rough estimates of when we expect parasites to have reached the 50 mg threshold and host manipulation to set in (Figure S2). Parasites in fish only infected on day 0 should have reached the 50 mg threshold just over 60 days post infection (i.e. around time point 2) and should hence have manipulated their host from time point 2 or 3 onwards. Parasites in fish only infected on day 31 should have reached the 50 mg threshold around day 50 post infection (i.e. around day 80 after the first infection, between time point 3 and 4). Accordingly, parasites from day 31 should only manipulate actively during the fourth time point. For sequentially infected fish, manipulation should depend on the outcome of a conflict over when manipulation should set in between the parasite from day 0 and the parasite from day 31 sharing a host. The parasite from day 0 in sequential infections reached 50 mg around 60 days post infection, around the same time as parasites from day 0 that did not have to share their host. If it dominates, manipulation should set in during time point 2 or 3. By contrast, parasites that infected hosts already infected on day 0 on day 31 (sequential infections) failed to reach the 50 mg threshold before the end of the experiment. If the parasite from day 31 dominates host behavior, fish sequentially infected by one parasite on day 0 plus one on day 31 should be manipulated throughout the experiment. Of course, any intermediate behavior indicative of a compromise might also be possible. In either case, if true host manipulation is at work, sequentially infected fish should not be manipulated sooner or more strongly than fish only infected on day 0. Any such host manipulation would indicate an effect of energy drain rather than true host manipulation.

Surprisingly, fish infected on day 31 start displaying more risk prone behavior already from the second time point onwards when their parasite is just over 30 days old and not yet infective and thus should not induce risk prone behavior. During the same time also fish infected on day 0 (whose parasites accordingly are 31 days older) increase their risky behavior. However, their parasites are infective. When fish experience the heron during the second time point, they have already experienced it once before and might be aware that it does not occur a second time during the trial. This could have reduced the frightening level of this stimulus. We do see no evidence of any habituation in uninfected fish. However, infected fish are faced with an additional energy drain, which could cause them to habituate more readily to a frightening stimulus; they might learn more quickly to adjust their behavior because they have to eat more. This effect could be independent of parasite size, if energy drain rather than actual host manipulation is responsible for the behavioral changes we observed. Further similarities between fish infected on day 0 and on day 31 could be facilitated by differences in parasite growth. Parasites from day 31 grow faster (they become infective about 50 days after infection while parasites from day 0 only become infective just over 60 days post infection, see above, Figure S2) and hence they might drain more energy. Both of these explanations are consistent with behavioral changes caused by enhanced energy drain but not with behavioral changes caused by true host manipulation. True host manipulation will not enhance the risk taking of fish infected on day 31 during the second time point when their parasites are not yet infective.

### Parasite weight and parasite index after dissection

For both experiments we measured fish and parasite weight (Figure S3 A, B) and fish weight at dissection. From these we calculated the parasite index (parasite weight/combined fish and parasite weight) as a measure of relative parasite burden. We combined data of infected fish from experiment 1 and 2 and used an analysis of variance model (aov, R (R Development Core Team 2010)) with parasite index or parasite weight as response and the treatment (experiment 1) or the time (experiment 2) as independent variable followed by a Tukey’s post hoc test. Our treatment/ time significantly affected parasite index (F_4,108_=128, p<0.0001) and weight (F_4,109_=57, p<0.0001). While late fish, i.e. fish with infective parasites from experiment 2 harbored about the same total parasite weight as fish infected on day 0 and fish infected on day 0 plus on day 31 in experiment 1, they had a significantly lower parasite index (table S2, Figure S3) because their fish were much larger. This probably contributed to differences in stickleback’s risk averseness between experiment 1 and II.

**Table S2: Results of the Tukey post hoc tests for parasite weight and parasite index.** 1_1 fish sequentially infected by one *S. solidus* on day 0 plus one on day 31, 1_0 fish only infected by one S. solidus on day 0, 0_1 fish only infected by one S. solidus on day 31, early: fish harboring not yet infective parasites, late: fish harboring infective parasites.

| Parasite  Parasite weight  index | | | Experiment | | | | |
| --- | --- | --- | --- | --- | --- | --- | --- |
|  |  |  | I | | | II | |
|  |  |  | 1_1 | 1_0 | 0_1 | early | late |
| Experiment | I | 1_1 |  | 0.012 | <0.001 | <0.001 | 0.303 |
|  |  | 1_0 | 0.004 |  | <0.001 | <0.001 | 0.853 |
|  |  | 0_1 | <0.001 | <0.001 |  | <0.001 | <0.001 |
|  | II | early | <0.001 | <0.001 | <0.001 |  | <0.001 |
|  |  | late | <0.001 | <0.001 | 0.522 | <0.001 |  |

**Figure S3: Worm weight (A, B) and parasite index (C, D).** Treatment: 1_1 fish sequentially infected by one *S. solidus* on day 0 plus one on day 31, 1_0 fish only infected by one *S. solidus* on day 0, 0_1 fish only infected by one *S. solidus* on day 31. Time: early: fish harboring not yet infective parasites, late: fish harboring infective parasites. Horizontal grey lines indicate the critical size (see Tierney & Crompton, 1992) to reach infectivity (A, B) or the parasite index at which this critical size should have been reached given the average size of fish in that experiment (C, D).

## Supplementary information III: Differences in the reaction to the simulated heron attack

To test the fish’s reaction to the simulated heron attack, used Fisher's Exact Test in R (R Development Core Team 2010). We categorized each fish’s reaction into 5 different categories (None: Fish showed no reaction to the simulated heron attack, Slow movement: Fish moved slowly; Fast, erratic movement: Fish darted into any direction without reaching hiding or jumped around without obvious direction, Freezing: Fish remained motionless in its current position for at least several seconds; Fleeing to hiding: Fished moved into hiding within a few seconds after the simulated heron attack.) and analyzed the number of fish in each treatment that performed each reaction for each time point. Fish that were hiding during the simulated heron attack were excluded from the analysis. If we found significant differences, we conducted post-hoc tests by repeatedly conducting the same models on only two treatments for each possible treatment combination and used Bonferroni corrections to correct for multiple testing.

In experiment 1, treatment did not affect how fish reacted to the simulated heron attack during the first two time points (Fisher’s exact test: Time point 1: p=0.3882, Time point 2: p=0.1599, p=0.06143, Figure S4 A). During the third time point, sequentially infected fish tended to show less strong reactions such as no reactions at all or slow movements than uninfected fish that froze or hid more often (p adjusted (Bonferroni correction) = 0.087, Figure S4 A). During the fourth time point, treatment did have a significant effect on host behavior. Post hoc tests with Bonferroni corrections revealed that uninfected fish showed arguably more risk averse reactions than fish infected on day 0 (p adjusted=0.045, Figure S4 A) and fish sequentially infected on day 0 plus day 31 (p adjusted=0.006, Figure S4 A). Sequentially infected fish also behaved significantly different, arguably less risk averse from those infected only on day 31 (p adjusted=0.025, Figure S4 A).

In experiment 2, nearly all fish reacted to the simulated heron attack by fleeing to hiding. Accordingly, we found no significant differences between infected and uninfected fish (Fisher’s exact test: Early fish: p= 0.6342, Late fish 2: p= 0.7860, Figure S4 B).

**Figure S 4: Reaction to the simulated heron attack by treatment.** A: Experiment 1, B: Experiment 2. None: Fish showed no reaction to the simulated heron attack, Slow movement: Fish moved slowly; Fast, erratic movement: Fish darted into any direction without reaching hiding or jumped around without obvious direction, Freezing: Fish remained motionless in its current position for at least several seconds; Fleeing to hiding: Fished moved into hiding within a few seconds after the simulated heron attack. Treatment: 1_1 fish sequentially infected by one *S. solidus* on day 0 plus one on day 31, 1_0 fish only infected by one *S. solidus* on day 0, 0_1 fish only infected by one S. solidus on day 31, 0_0 uninfected fish. N: Time point1: 1_1: 24, 1_0: 30, 0_1: 19, 0_0: 35; Time point2: 1_1: 24 , 1_0: 29, 0_1: 18, 0_0: 35; Time point3: 1_1: 26, 1_0: 29, 0_1: 18, 0_0: 33; Time point4: 1_1: 26, 1_0: 29, 0_1: 19, 0_0: 31.

**References for supplementary information**

Barber I, Svensson PA. 2003. Effects of experimental *Schistocephalus solidus* infections on growth, morphology and sexual development of female three-spined sticklebacks, *Gasterosteus aculeatus*. Parasitology 126:359–367.

R Development Core Team. 2010. R: a language and environment for statistical computing. R Found. Stat. Comput.

Scharsack JP, Kalbe M, Derner R, Kurtz J, Milinski M. 2004. Modulation of granulocyte responses in three-spined sticklebacks *Gasterosteus aculeatus* infected with the tapeworm *Schistocephalus solidus*. Dis. Aquat. Organ. 59:141–150.

Scharsack JP, Koch K, Hammerschmidt K. 2007. Who is in control of the stickleback immune system: interactions between *Schistocephalus solidus* and its specific vertebrate host. Proc. R. Soc. B 274:3151–3158.

Tierney JF, Crompton DWT. 1992. Infectivity of plerocercoids of *Schistocephalus solidus* (Cestoda: Ligulidae) and fecundity of the adults in an experimental definitive host, *Gallus gallus*. J. Parasitol. 78:1049–1054.
